# Burnei-Gavriliu classification of congenital scoliosis

**Published:** 2015

**Authors:** RA Ghiță, I Georgescu, ML Muntean, Ș Hamei, EM Japie, C Dughilă, I Țiripa

**Affiliations:** * Department of Pediatric and Orthopedic Surgery, “Maria Sklodowska Curie” Emergency Hospital for Children, Bucharest, Romania; ** Baneasa Hospital – “Regina Maria” Private Health Care Network, Bucharest, Romania; *** Department of Pediatric Surgery, Brasov, Romania

**Keywords:** congenital scoliosis, classification, rotational imbalance, longitudinal imbalance, hemivertebra

## Abstract

**Introduction:** The existent classifications of congenital scoliosis cannot contain all the cases encountered in the medical practice taking into account the complexity of the spine deformity in this pathology.

**Purpose:** The paper represents a retrospective study that analyses a new classification of congenital scoliosis in comparison with the existing classification.

**Materials and method:** This study analyses 56 cases over a period of 14 years (2000-2013), based on the spine dominant deviation: longitudinal or rotational imbalance.

**Results:** This new classification contains not only the formation, segmentation and mixed defects, but also the formation failure with or without fusion failure of the ossification centers (wedged vertebra, hemivertebral body, segmented, hemisegmented, unsegmented hemivertebra, which may be successive, intermittent, alternant compensated or alternant decompensated). It also contains the congenital scoliosis with a rotational imbalance by spinal traction, spinal pushing or by a mixed effect.

**Discussion:** In comparison with the other classifications in literature: Winter, Imagama or Kawakami, this classification systematizes data according not only to the spatial disposition of the hemivertebrae, but also to the balance of the deformity.

**Conclusions:** In conclusion, this classification of congenital scoliosis has a practical, diagnostic, therapeutical and prognostic use.

## Introduction

Congenital scoliosis is a complex malformation of the spine, which is usually associated with other abnormalities, creating many problems for the doctor, the patient and the patient’s family. In practice, this deformity may be also approached from both the deformity’s severity and treatment indication point of view. The classification of congenital scoliosis has a great significance regarding this aspect.

The medical purpose is to obtain a spine without any pelvic or shoulders imbalance with no neurologic deficiency at the end of the growth period. Basically, the success of the treatment success is obtained if the diagnosis is early established, before its progression into a severe, stiff and large curve. Basic scientific researchers have established as risk factors, clinically difficult to establish, the exposure of the pregnant woman to carbon monoxide, the diabetes mellitus in pregnant woman and the administration of anti-epileptic drugs in pregnant woman [**1**-**3**]. The genetic transmission is for now only presumed, taking into account the multitude of the associated malformations [**4**] or the presence of the malformations within some diseases that are certainly transmitted genetically [**5**].

Congenital scoliosis may show segmental malformations disposed at different levels. If they are disposed on both sides, the spine can be balanced with or without a scoliotic curve.

## Classification

As a concept, the classification of congenital scoliosis should contain the embryological, anatomopathological, biomechanical, evolution and therapeutic potentials data inside each diagnosis.

The first congenital scoliosis classification was made by Winter and collaborators in 1968 studying a series of 234 patients [**6**]. This classification stands on the radiographic findings. The types of malformations described by Winter are in agreement with the following definition of congenital scoliosis, defined as a formation, segmentation or mixed defect (**Table 1**). The balanced scoliosis represents around 26% of the congenital scoliosis and the surgical intervention is not necessary: 11% of these do not develop in time and 15% show a minimal deformation. For the most part, 74% of the cases, the congenital scoliosis advances, resulting a notable deformation associated with cardiac and respiratory disorders, which need surgical intervention [**7**].

**Table 1:** Winter et al. classification

	Complete failure – hemivertebra, butterfly vertebra
Failure of formation	Partial failure – wedged vertebra
	Unilateral failure – longitudinal bar
Failure of segmentation	Bilateral failure – block vertebra
Miscellaneous	Formation and segmentation mixed failure

In 2004, Imagama and collaborators [**8**,**9**] elaborated a classification of congenital scoliosis which took into account the formation errors and the structural combining of the anterior and posterior vertebral component. The study analyzed a series of patients with congenital spine malformations and by means of a 3D CT brought into the spotlight the deformity association of the two components, guiding the surgeons through the complexity of the malformations lesions (**Table 2**).

**Table 2:** Imagama et al. classification

Anterior component	Posterior component
Hemivertebra (hemi-pedicle)	Fully segmented hemilamina
	Semisegmented hemilamina
	Spina bifida
	Bilamina (complete or incomplete)
Butterfly vertebra (bipedicle)	Wedged lamina
	Spina bifida
Lateral wedged vertebra (bipedicle)	Wedged lamina

In 2009, Kawakami [**10**,**11**] classified the congenital scoliosis in a 3D CT study on a series of 150 patients, in 4 types of anomalies: solitary simple, multiple simple, complex and pure segmentation failure (**Table 3**).

**Table 3:** Kawakami et al. classification

Type 1	Solitary simple congenital malformation (unison)
	• hemivertebra
	• wedged vertebra
	• butterfly vertebra
	• others
Type 2	Multiple simple anomalies (unison)
	• combination of hemivertebra, wedged vertebra and butterfly vertebra
	• discreet, adjacent or others
Type 3	Complex anomalies (discordant)
	• mixed failure
Type 4	Segmentation failure only

In 2014, 56 cases of congenital scoliosis were analyzed in “Professor Alexandru Pesamosca” Clinic of “Maria Sklodowska Curie” Children’s Emergency Hospital Bucharest. The patients were observed in our clinic from 2000 to 2013. 42 of these patients underwent surgical interventions using several procedures. The statistical analysis and the type of malformation did not allow us to include all the encountered anomalies within the mentioned classifications. The cases classified in the known literature database and the necessity to classify the other cases led to a new classification consisting of two groups based on the spine dominant deviation in coronal and transverse view: scoliosis due longitudinal and rotational imbalance (**Table 4**).

**Table 4:**
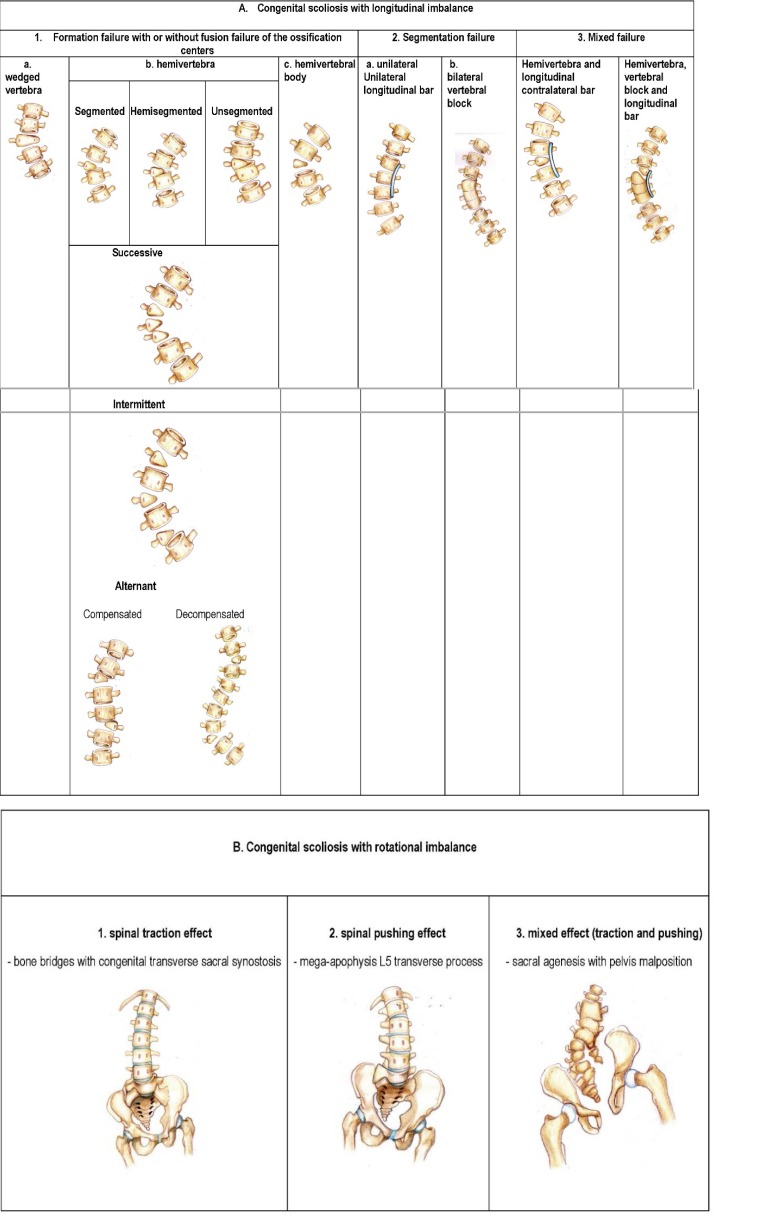
Burnei-Gavriliu classification

## Discussions

The congenital scoliosis with longitudinal imbalance has as anatomopathological substratum the formation, segmentation and mixed failure.

**Fig. 1:**
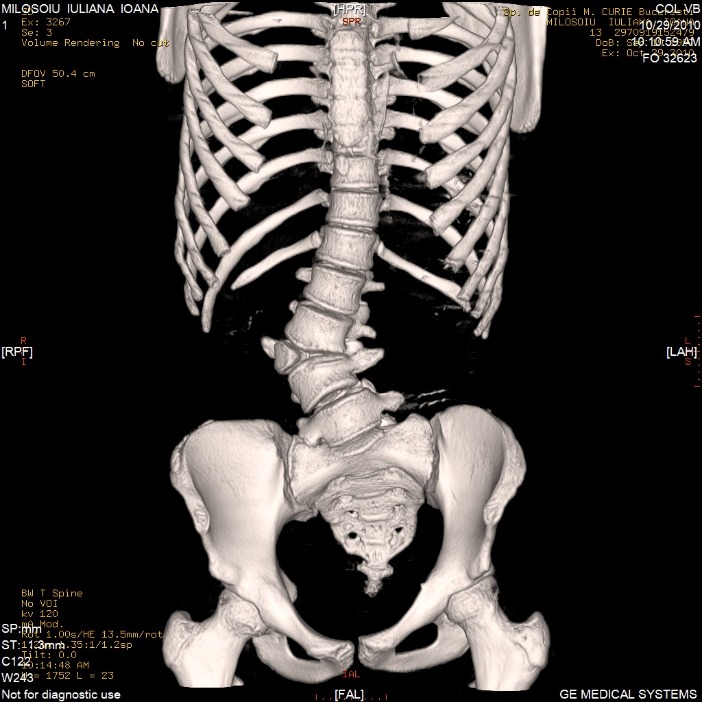
L3 segmented hemivertebra

*The defect of formation* consists of a wedged vertebra with one of the somatic halves hypoplastic with a smaller pedicle than the opposite one. The defect of formation may also consist of a hemivertebra with only one pedicle (**Fig. 1**). 

**Fig. 2:**
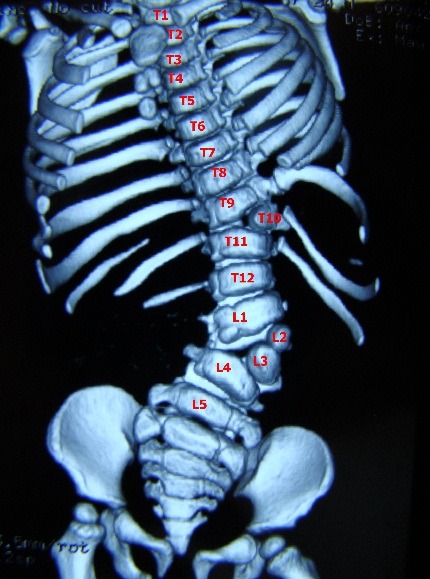
One intermittent hemivertebra and 2 successive hemivertebrae: T10 – left segmented hemivertebra with hypotrophic pedicle and transverse apophysis, a L2 vertebral hemibody and a L3 fully segmented hemivertebra

The hemivertebra may be segmented, hemisegmented or unsegmented. The segmented hemivertebra has an intervertebral disc above and beneath it; the hemisegmented has only one disc, the other surface consisting of a fibrous lamellar tissue. The unsegmented hemivertebra has a fibrous lamellar tissue above and beneath it. There are cases with more than one hemivertebra that may be situated on the same side or bilaterally. When they are placed on the same side, we may have successive hemivertebrae or when the hemivertebrae are intercalated with the normal vertebrae, we may have intermittent hemivertebrae (**Fig. 2**). 

**Fig. 3:**
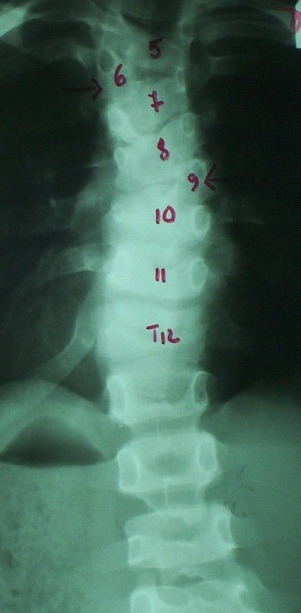
Compensated congenital scoliosis – 2 alternant hemivertebrae: T6 and T9 with 2 normal vertebrae between them

**Fig. 4:**
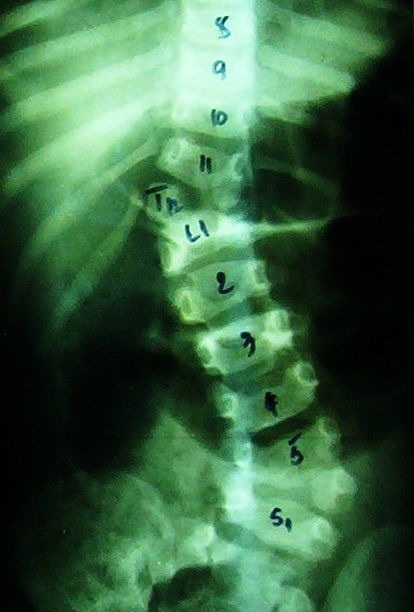
Decompensated congenital scoliosis – 2 alternant hemivertebrae: T12 and L5’ with 5 normal vertebrae between them

If they are situated alternant bilaterally, we may talk about a double scoliosis that can be compensated, when less than 5 normal vertebrae (**Fig. 3**) are situated between the 2 hemivertebrae, or decompensated, when there are more than 5 normal vertebrae (**Fig. 4**) between the 2 hemivertebrae.

*The defect of segmentation* consists of a bar situated on the concave side of the spine for a distance of 2, 3 or even more vertebrae. Another defect of segmentation is the circumferentially synostosis or synchondrosis of 2 or more vertebrae that may be symmetrical (brachy-spondylitis) or asymmetrical (vertebral block).

**Fig. 5:**
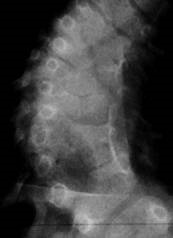
Hemivertebrae, vertebral block and longitudinal bar

*The mixed* failure consists of a bar on one side, associated with one or more segmented hemivertebrae on the opposite side (**Fig. 5**). This type of failure has the highest evolution potential, and that is why, a correct evaluation and a surgical intervention at the proper time are indicated in order to avoid the possible serious complications. 

**Fig. 6:**
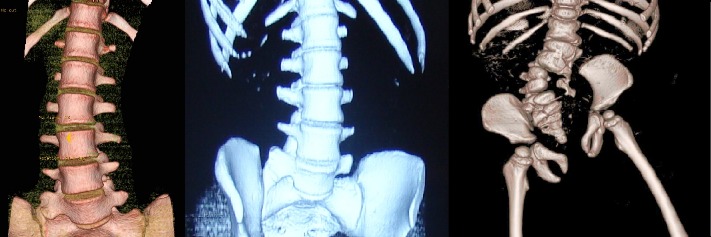
Rotational imbalance: a. Traction effect, b. Impingement effect, c. Mixed effect

The vertebral rotation in congenital scoliosis with rotational imbalance prevails against the coronal deviation. These types of scoliosis are secondary to a vertebral and/ or a pelvic congenital malformation, which has as a main effect the vertebral rotation by traction, pushing or mixed (**Fig. 6**). Usually, the scoliosis and vertebral rotation are not present at birth. Initially, the vertebral rotation and gait problem appears during growth and development period, followed by the presence of a scoliotic curve.

The evolutive potential depends on the type of deformity. Compared to a normal growth of the spine [**12**], the appearance and evolution of the curve are due to the asymmetric growth on the convex side in comparison to the concave one. A grading of the curve prognosis looks as it follows [**13**]: 

1. Fully segmented hemivertebra (two growth plates) with a contralateral bar that can get worse in the following 10 years

2. Unilateral unsegmented bar

3. Two consecutive fully segmented hemivertebrae

4. Fully segmented hemivertebra

5. Hemisegmented hemivertebra

6. Wedge vertebra (corresponding to a partial failure of formation)

7. Incarcerated hemivertebra (these are smaller and they induce little deformity as the hemivertebra sits in a non-growing block where the hemivertebra has created a niche; the alignment of the pedicles usually remains straight).

The concepts regarding the treatment are in a continuous change trying to be adapted to each type of deformity. Nowadays, the surgeon does not have to choose between a “long”, but curved spine or a “short”, but straight spine. It is generally accepted that no treatment cannot completely correct the effects of a congenital error that appears in the spinal configuration [**14**-**16**]. That is why the treatment has to be adapted to each type of deformity trying to maintain a growing and development of the spine as normal as possible.

## Conclusions

In comparison with the above-mentioned classifications, Burnei-Gavriliu classification is broader: it includes the Winter classification, it systematizes data according to the spatial disposition of the hemivertebrae, the balance of the deformity, and adds the scoliotic deviations induced only by the presence of the hemivertebral body.

In addition to the defining elements of the defects of formation and segmentation types, it also includes some other rare cases that cannot be included in the existing classifications. This classification has a practical, diagnostic, therapeutical and prognostic use, based on the imagistic study.

The classification may be labeled as imagistic, based on embryological, anatomopathological and biomechanical data and having an appreciative value on the evolution potential and the therapeutic approach.
